# Supplementary material for: The causal role of circulating inflammatory markers in osteoporosis: a bidirectional Mendelian randomized study
Source: Front Immunol. 2024 Jul 18;15:1412298. doi: 10.3389/fimmu.2024.1412298 (PMC11291241; doi:10.3389/fimmu.2024.1412298)
Supplement: Supplementary file 1 [file DataSheet_1.zip › supplementary material/CIM-OSTEOPOROSIS /GCST90274817.csv_forest.pdf]

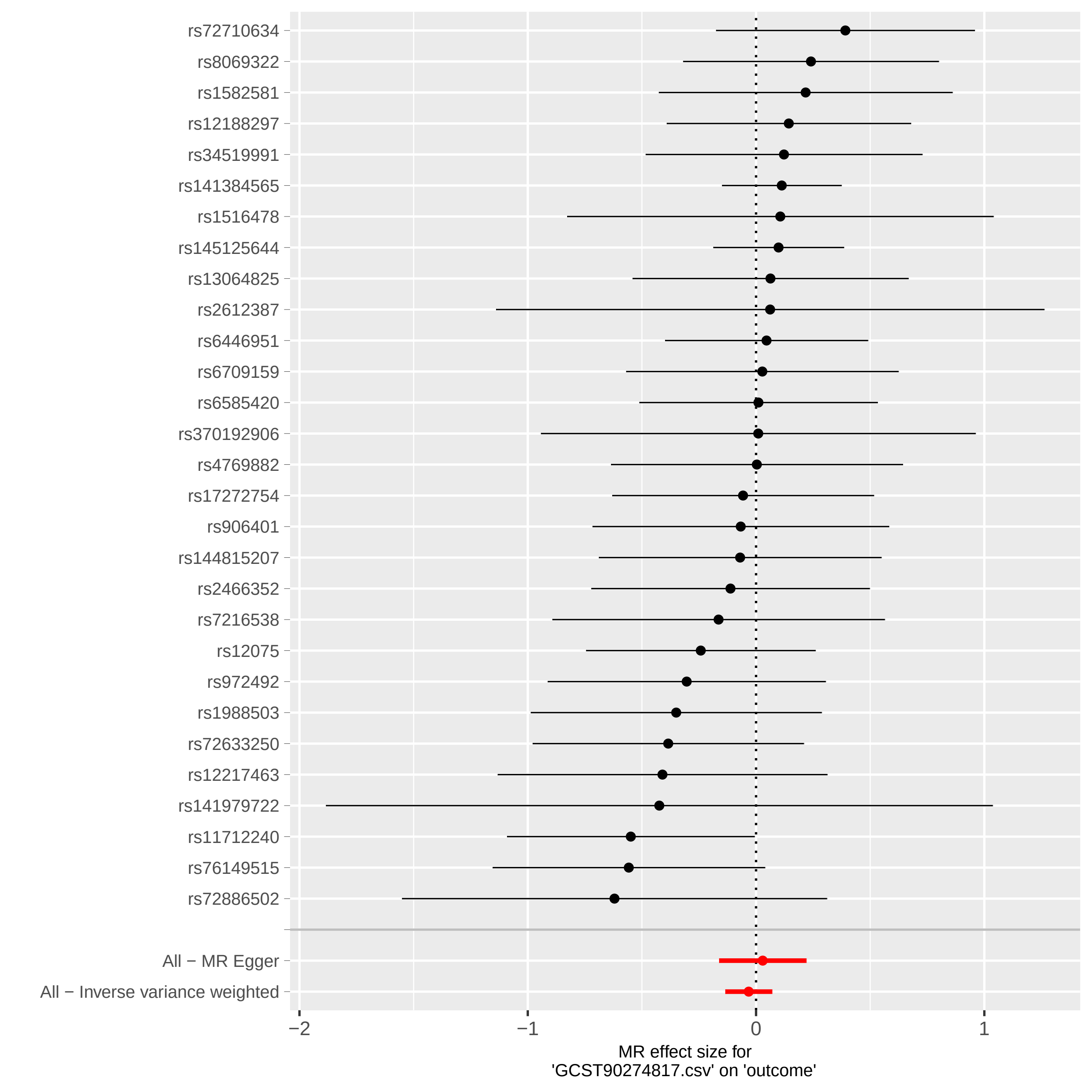

rs72710634
rs8069322
rs1582581
rs12188297
rs34519991
rs141384565
rs1516478
rs145125644
rs13064825
rs2612387
rs6446951
rs6709159
rs6585420
rs370192906
rs4769882
rs17272754
rs906401
rs144815207
rs2466352
rs7216538
rs12075
rs972492
rs1988503
rs72633250
rs12217463
rs141979722
rs11712240
rs76149515
rs72886502
All − MR Egger
All − Inverse variance weighted
−2
−1
0
1
MR effect size for
'GCST90274817.csv' on 'outcome'
